# Ethnic disparities in clinical severity of coronavirus disease 2019 (COVID-19) upon admission

**DOI:** 10.1093/eurpub/ckac131.524

**Published:** 2022-10-25

**Authors:** M Goldschmidt, M Norredam, T Benfield

**Affiliations:** 1 Centre for Migration, Ethnicity and Health, Institute of Public Health, Copenhagen University, Copenhagen, Denmark; 2 Department of Infectious Diseases, Hvidovre Hospital, Copenhagen, Denmark

## Abstract

**Background:**

Reports from all over the world have shown great ethnic disparities in COVID-19 morbidity and mortality. Recent European studies have found discrepancies between higher risk of admission and intensive care treatment among ethnic minorities but lower mortality rates compared to the ethnic majority. This study will elucidate the ethnic differences in disease severity upon admission as a possible factor in explaining these discrepancies.

**Methods:**

A retrospective cohort study of 1442 patients admitted with COVID-19 at three hospitals in Copenhagen, Denmark, between 1st February 2020 and 31st May 2022. Clinical, demographic and ethnicity data were extracted from health care records and collected using REDCap. Severity upon admission (< 24 hours) was assessed as 1) oxygen need, 2) oxygen administration and 3) need for intensive care.

**Results:**

Ethnicity was registered on 1341 patients (57,0% Danish, 34,8% non-Western). Over all, preliminary descriptive analyses show patients of non-Western origin had symptoms of COVID-19 for a longer period (8,0 vs 6,7 days, p < 0,0001) and had a higher oxygen need (7,0L vs 5,2L, p = 0,02) upon admission compared to patients of Danish origin. A higher percentage of patients of non-Western origin needed high flow oxygen administration upon admission (30,2% vs 22,9%, p = 0,006) and were transferred to the ICU within the first 24 hours (4,9% vs 2,2 %, p = 0,02) compared to patients of Danish origin. Further analysis will be done, including biochemistry and link to registers in order of obtaining more accurate info on country of birth and migration status. We will do logistic regression regarding ethnic differences in severity of COVID-19 upon admission adjusting for comorbidities, age, sex and BMI.

**Conclusions:**

Preliminary data on disease severity of COVID-19 upon admission show some ethnic disparities. Language barriers, low health literacy or the fear of stigma might explain this. Further analyses are needed.

**Key messages:**

• Preliminary data from this cohort study suggest that patients of non-Western origin had symptoms of COVID-19 for a longer period before being admitted compared to patients of Danish origin.

• Preliminary data on disease severity of COVID-19 upon admission show ethnic disparities with regard to oxygen need, oxygen administration and need for intensive care.

